# Development and application of vibrating dynamic culture system for mouse oocytes and embryos

**DOI:** 10.3389/fvets.2025.1606746

**Published:** 2025-07-08

**Authors:** Qinli Liu, Sen Zhao, Jian Zhou, Yu Shi, Chongyang Ye, Bo Huo

**Affiliations:** ^1^Department of Mechanics, School of Aerospace Engineering, Beijing Institute of Technology, Beijing, China; ^2^Reproductive Medical Center, Amcare Women's and Children's Hospital, Tianjin, China; ^3^College of Arts, South China Agricultural University, Guangzhou, Guangdong, China; ^4^School of Fashion and Textiles, The Hong Kong Polytechnic University, Kowloon, Hong Kong, SAR, China; ^5^Sport Biomechanics Center, Institute of Artificial Intelligence in Sports, Capital University of Physical Education and Sports, Beijing, China

**Keywords:** dynamic culture, parthenogenetic activation, compaction, fluid shear stress, finite element method

## Abstract

**Background:**

Mammalian oocytes fertilization and early embryos development primarily take place in the fallopian tube, which not only provides nutrients but also offers a suitable mechanical environment. The current culture system for oocytes and embryos in assisted reproductive technology is static, leading to weak developmental potential and an implantation rate of only 30%-40%. It is speculated that the low developmental potential may be due to the significant difference between the static culture method and the *in vivo* dynamic mechanical environment of the embryos. However, the mechanisms through which mechanical stimulation affects the *in vitro* maturation of oocytes and early embryos development remain unclear. This study aimed to investigate how vibrational stimulation affects both nuclear maturation efficiency and the subsequent parthenogenetic developmental competence of mouse oocytes.

**Materials and methods:**

This study designed and fabricated a vibration loading device that simulates the *in vivo* mechanical environment of the fallopian tube. Furthermore, a numerical simulation was performed to study the effects of different loading parameters (vibration frequency and vibration amplitude) on the fluid shear stress (FSS) in the device. Immature mouse oocytes were cultured in static or vibrating (3 Hz, 6 Hz, or 10 Hz) conditions. The maturation rate, embryos compaction rate and formation rate of parthenogenetic blastocysts were compared.

**Results:**

The numerical simulation results showed that the average wall fluid shear stress was 0.09-3.2 dyne/cm^2^ when the vibration frequency was 3–10 Hz and the vibration amplitude was 0.1–1 mm. The experiment results indicate that mechanical stimulation had no significant effect on the *in vitro* maturation of immature mouse oocytes compared with the static culture group. However, mechanical loading at 3 Hz, 6 Hz, and 10 Hz vibration (0.1 mm amplitude), and 3 Hz vibration (1 mm amplitude) significantly increased embryo compaction, and improved the blastocyst formation rate, thereby enhancing the developmental potential of immature mouse oocytes.

**Conclusions:**

This study developed a vibration device to simulate the *in vivo* mechanical environment. The loading parameters were predicted using numerical simulations, and the experiment results showed that when the wall fluid shear stress exceeded 2.0 dyne/cm^2^, embryonic development potential was significantly reduced. This study provides a dynamic culture device for clinical assisted reproduction and contributes to understanding the regulatory effects and mechanisms of mechanical stimulation on the *in vitro* maturation of immature oocytes and embryonic development.

## Introduction

In the process of assisted reproductive technology, *in vitro* fertilization involves ovarian hyperstimulation to obtain oocytes. Among these, 10%−20% of oocytes are immature. After *in vitro* maturation, these immature oocytes generally exhibit poorer quality compared to *in vivo-*matured oocytes, resulting in lower developmental potential. Edwards conducted significant research on the *in vitro* maturation of human oocytes in the 1960s (1). Cha et al. reported the first birth from an immature oocyte *in vitro* maturation cycle (2). The recognized advantages of oocyte *in vitro* maturation include avoiding the need for high doses of gonadotropins to stimulate the ovaries, thereby reducing costs and preventing the occurrence of ovarian hyperstimulation syndrome (OHSS), making it a relatively safe treatment option (3). It is mainly applied in the treatment of infertility in patients with polycystic ovary syndrome (PCOS), high-responding ovarian cases, and cancer patients (4). To date, over 5,000 babies have been born from *in vitro* maturation cycles worldwide, however, the clinical pregnancy rate remains low, around 20% to 30%, which is lower than the pregnancy rate from embryos derived from mature oocytes. There are several problems with the current oocyte *in vitro* maturation technology, including low maturation rates, asynchrony between nuclear and cytoplasmic maturation of oocytes, and reduced developmental potential of the resulting embryos (5). These issues indicate that further optimization of oocyte *in vitro* maturation technology is necessary.

With advancements in assisted reproductive technology, researchers are increasingly focusing on the delicate and complex physiological functions of the fallopian tubes. As oocytes and embryos pass through the fallopian tubes, they are subjected to mechanical stimulus (6, 7). The mechanical effects of transporting oocytes and embryos through the fallopian tubes are reflected in a combination of factors such as muscles, cilia, and fallopian tube secretory cells, which work together to produce forces that move or fix eggs and embryos (8, 9). Therefore, the growth and development of early embryos are carried out in a dynamic mechanical environment. The current culture system for oocytes and embryos in assisted reproductive technology is static. This static culture method results in weak developmental potential, leading to an implantation rate of only 30%−40% (10). It is speculated that the low developmental potential may be due to the significant difference between the static culture method and the dynamic mechanical environment of the embryo *in vivo*. So, how to provide a suitable mechanical environment for embryos is one of the urgent problems to be solved.

The role of the physical environment (e.g., mechanical environment and substrate surface topography) to which the early embryo exposed has been relatively little studied. Hoe et al. found that a dynamic micro-funnel culture system significantly increased the blastocyst formation rate in mice (11). Hara et al. found that the tilted dynamic culture system significantly improves the development of mouse and human embryos to blastocysts (12). Micro-vibrational stimulation of cilia can also significantly enhance the developmental potential of mammalian embryos (8, 13, 14). Altogether, these studies suggest that providing gentle mechanical stimulation of early embryos by simulating the oviduct environment can promote early embryo development.

Current research indicated that the use of these dynamic culture devices can enhance the *in vitro* developmental potential of embryos. However, the vibration culture devices used in these studies have employed vibration frequencies (20–80 Hz) that exceed the physiological range (distribution range of vibration frequency is 3.4-20 Hz), and the vibration was applied in a non-continuous manner, which was inconsistent with physiological conditions. In this study, we investigated the effects of different vibration frequencies (3 Hz, 6 Hz, and 10 Hz) and amplitudes on the *in vitro* development of immature oocytes. At the same time, we also used numerical simulation methods to analyze the effects of vibration frequency and amplitude on the fluid shear stress and equivalent strain experienced by cells, thereby identifying an optimal vibration range for oocyte cultures. Therefore, we investigated the effects of different vibration frequencies and amplitudes on the *in vitro* development (maturation rate and the subsequent parthenogenetic developmental competence) of immature oocytes in this study.

## Materials and methods

### Oocytes and embryos culture

With the approval of the Animal Ethics Committee of Institute of Radiation medicine of Chinese, Academy of Sciences & PEKING Union Medical College. The animal use in this research has been reviewed and approved by the Animal Ethical and Welfare Committee (AEWC). 6- to 8-week-old SPF-grade CD1 female mice were purchased from Beijing Viton Lever Laboratory Animal Technology Co. The mice were injected with pregnant mare's serum gonadotropin (PMSG) at a dose of 5 IU per mouse, and 42~46 h later, mice were sacrificed by cervical dislocation. The mice were gently restrained, and cervical dislocation was performed by experienced personnel. This involved firmly grasping the head and the trunk of the mouse and applying a sudden, well-controlled force to separate the cervical vertebrae, leading to instantaneous death. The ovaries were removed for germinal-vesicle (GV) stage oocytes collection. The immature oocyte-cumulus complex appears dark with a dense radiating corona and a granulosa cell cluster tightly attached to zona pellucida. The cytoplasm contains an intact germinal vesicle.

The collected immature oocytes were placed in an immature oocyte culture medium (95% MEM (Invitrogen, cat. No. 11095-080), 5% FBS (Gibco), 0.24 mM sodium pyruvate (Sigma), 1.5 IU/mL hCG, 1 IU/mL PMSG.) and cultured for 14–16 h. Oocytes were observed under an inverted microscope, and the first polar body was excluded to determine nuclear maturation. The oocytes were transferred into solution (80 IU/mL hyaluronidase in MII medium) to remove the granulosa cells. The clean oocytes were washed in potassium simplex-optimized medium (KSOM) 3 times and transferred to the parthenogenetic activation medium (Ca^2+^-free KSOM [the formulation details the previous studies (15)] with additional 10 mM SrCl_2_ and 2.5 μg/mL cytochalasin D) for 4 h. During parthenogenetic activation, all oocytes were cultured statically with no mechanical stimuli applied in either the dynamic or static culture groups. The activated oocytes were then transferred to KSOM for culture.

### Development of vibrating dynamic culture system

When oocytes and embryos pass through the fallopian tubes, they are influenced by a comprehensive mechanical stimulus such as cilia vibration inside the tubes, fluid flow in the fallopian tubes, and muscle contraction force. Cilia means vibrating hair in Latin. The distribution range of vibration frequency of cilia is 3.4–20 Hz in mammals.

**Figure 1:**
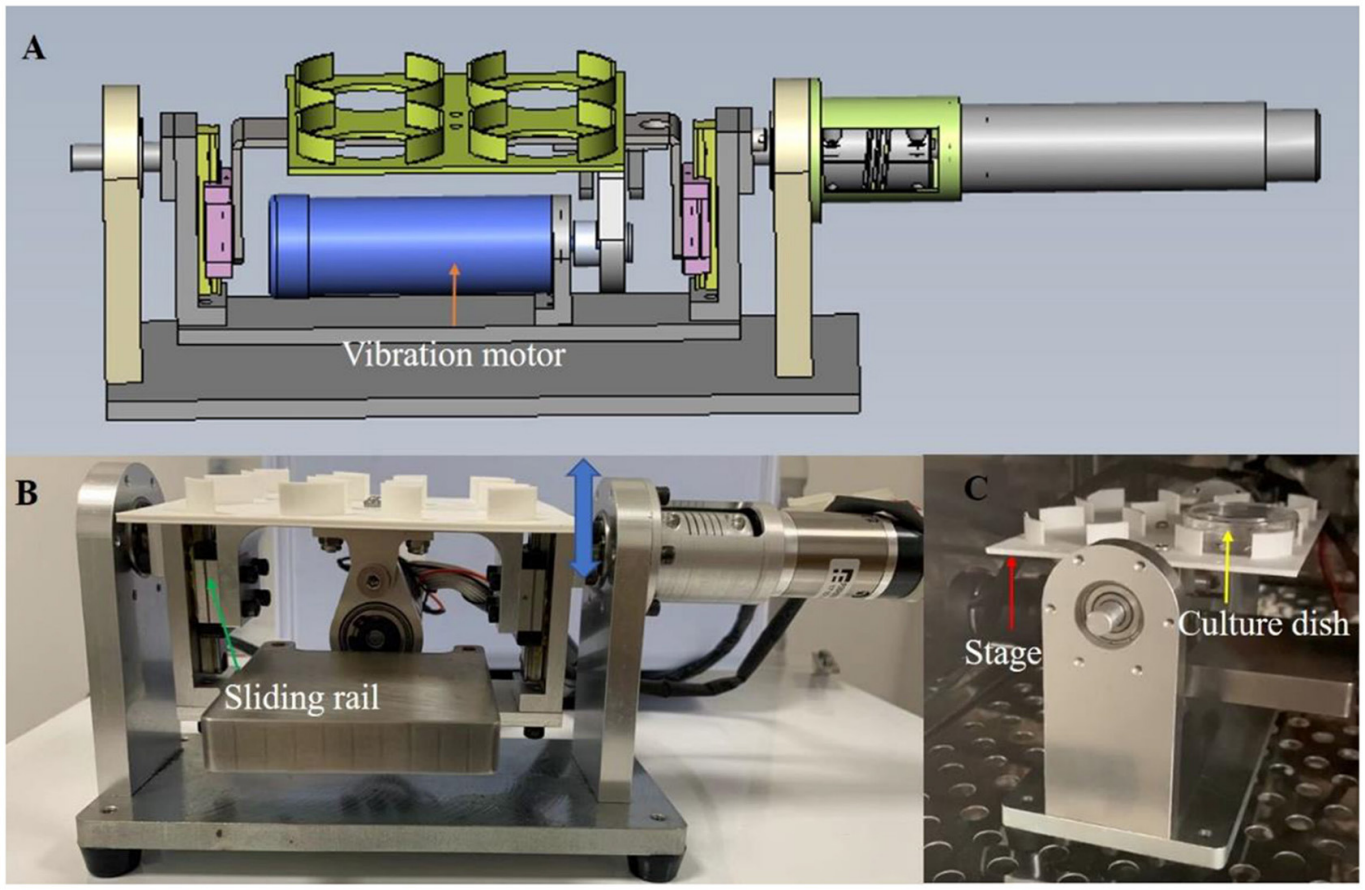
The custom-made vibration device providing vibration condition for embryo culture. **(A)** the schematic diagram of the device. **(B, C)** the photos of the device from frontal and side view. Culture dish (yellow arrow) is set on the stage (red arrow). The vibration motor drives the eccentric wheel movement, causing the sliding rail (green arrow) to move up and down (blue two-way arrow), thus causing the movement of the stage to realize the vibration stimulation. The device can be used in a humidified incubator **(C)** and include a waterproof vibration motor.

Based on the mechanical environment of the fallopian tube, this study designed and fabricated a vibrating dynamic device (Figure 1). The device consists of two parts: culture dish platform and vibration drive. The culture dish platform is located above the device. It holds the culture dishes. The vibration drive provides vertical vibrations to the culture dish platform at a specific frequency. The inner side of the device is equipped with a guide rail, and the vibration motor drives the guide rail to move up and down, thereby causing the culture dish platform to also move vertically, achieving the desired vibration. The frequency and amplitude of the vibrations can be adjusted as needed, with a frequency range of 0 to 50 Hz and an amplitude range of 0.1–1 mm.

### Application of vibrating dynamic culture system

Oocytes were randomly divided into traditional static culture and vibration culture group. Parameters such as the frequency and duration of the vibration can be adjusted. In this study, three vibration frequencies were set for the device to 3 Hz, 6 Hz, and 10 Hz with different vibration amplitude (0.1 mm and 1 mm) to investigate the effect of dynamic culture on the maturation and parthenogenetic developmental potential of immature mouse oocytes (vibrational cultured statically after maturation).

Due to the special and complex structure of the fallopian tubes, it is very difficult to measure the force exerted on oocytes and early embryos *in vivo*. Numerical simulation has become an effective method for studying the distribution of fluid shear stress (FSS) in the mechanical microenvironment of the fallopian tubes. Therefore, in order to predict the FSS and equivalent strain experienced by cells in a vibration mechanical loading device. This study conducts numerical simulations of the cells in this device using the finite element (FE) method.

### Numerical simulation to predict the FSS of vibrating dynamic culture system of cells

**Figure 2:**
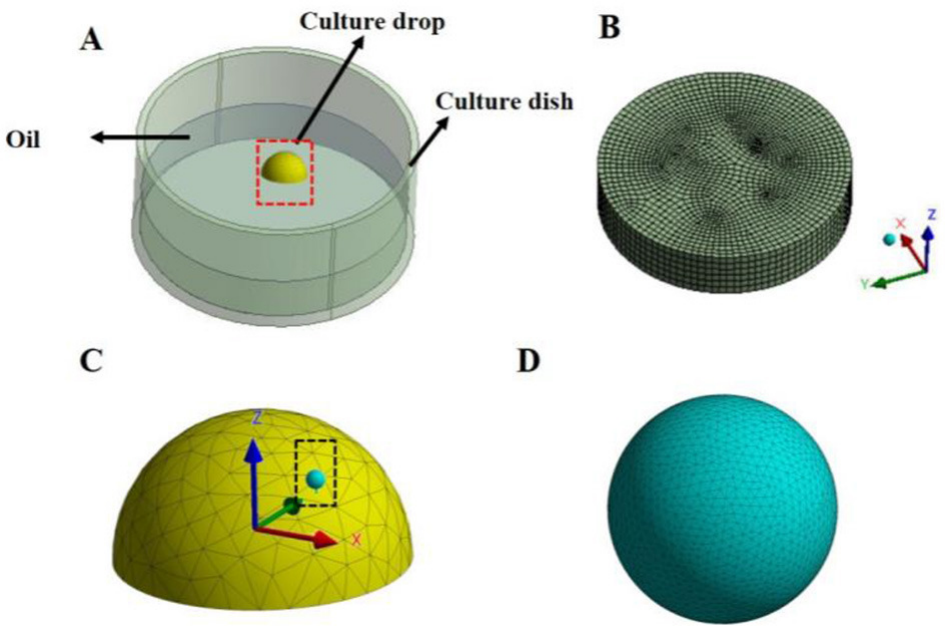
Finite element model of the cell loading device. **(A)** Geometric model of the culture dish: The diameter of the culture dish is 35 mm, and a 50 μL droplet of hemispherical culture medium is placed on the bottom surface, which is evenly covered by 3 mL of paraffin oil. In the culture medium droplet, cells with a diameter of 80 μm are placed; **(B)** Mesh diagram of the paraffin oil; **(C)** Magnified view of the red dashed box in diagram A, showing the mesh of the hemispherical droplet; **(D)** Magnified view of the black dashed box in diagram C, showing the mesh of the spherical cells in the droplet.

The geometry model of the vibrating dynamic culture was constructed by using Solidwork system 2018 software (Dassault Systemes, USA) based on the practical size with the paraffin oil, culture liquid, and 80 μm radius of cells as the flow domain (Figure 2). Navier-Stokes equations were used to define the flow behavior of viscous fluids. The culture liquid was assumed as incompressible viscous fluid with a density of 1 × 10^3^ kg/m^3^ and a viscosity coefficient of 1.7 × 10^−3^ Pa·s (16), and the paraffin oil was also assumed as incompressible viscous fluid with a density of 850 kg/m^3^ and a viscosity coefficient of 0.01 Pa·s (17).

The geometry model was meshed by linear Fluent tetrahedron element with 0.2 mm element size. The elements and nodes quantity are ~70,000 and 120,000, respectively. The constructed geometry model and mesh would be imported into ANSYS Fluent v19.2 (ANSYS, Pennsylvania, Pittsburgh, USA) to numerically simulate the oocytes FSS and culture fluid pressure.

For the boundary conditions, a free surface was used for the upper fluid surface within the well. The vibrating system was adopted as a moving wall boundary condition with the 3 Hz, 6 Hz, and 10 Hz vibration frequencies and 0.1 mm and 1.0 mm vibration amplitude, respectively. An iterative method was used to solve the equations for steady flow, and convergence was identified when the relative tolerance was <0.001.

### Numerical simulation to predict the stiffness of cells in the vibrating dynamic culture system

The geometry model of cells was constructed by using Solidwork system. The oocytes or the embryos can be assumed as 80 μm radius hemisphere. The constructed geometry model was imported into ANSYS workbench to predict the stiffness of cells in the vibrating dynamic culture system.

The geometry cells model was meshed with linear tetrahedron elements, and the elements' and nodes' number are approximately 32,000 and 51,000, respectively. The mesh quality was about 0.7, indicating good mesh quality applied in our developed FE model of cells.

The simulated FSS and culture fluid pressure was imported into ANSYS workbench as the boundary conditions. Von-Mises equivalent stress of the cells in the vibrating dynamic culture system can be predicted based on simulated FSS and culture fluid pressure.

The displacement support was adopted to restrict the cells vertically vibrate along the vibrating dynamic culture system. By assessing the freedom of the FE model of cells, the weak spring constraint was adopted on the cells surface to balance the stiffness matrix of the basic equation of elasticity machines after discretization, which applied to eliminate the oocytes stiffness deformation. The time steps were set as 1 s, and the step end time was set as steady state. The calculated Von-Mises stress and simulated Von-Mises strain was applied to estimate the oocytes stiffness.

### Statistical methods

In this experiment, numerical simulations were used to calculate the FSS experienced by cells under different loading conditions. Subsequently, cell experiments were conducted to observe how various loading parameters affect the developmental potential of the cells, thereby determining the suitable mechanical parameters for the device. In this study, SPSS 17.0 software (SPSS Inc. Chicago, IL, USA) was used for statistical analysis, the χ^2^ test was used to compare the count data. *P* < 0.05 indicated a statistically significant difference.

## Results

### Effect of dynamic culture on the *in vitro* maturation of immature oocytes

Eight hundred and ninety seven immature oocytes were cultured *in vitro*, of which 338 were static cultured, and 559 were cultured under vibration. Among them, 222, 185 and 152 immature oocytes were cultured in the 3 Hz, 6 Hz and 10 Hz vibration groups, respectively. The results showed that there were 295 (87.3%) mature oocytes in static culture group, 125 (94.7%), 72 (100%), 75 (97.4%), 82 (91.1%), 101 (89.4%) and 70 (93.3%) mature oocytes in 3 Hz (0.1 mm amplitude), 6 Hz (0.1 mm amplitude), 10 Hz (0.1 mm amplitude), 3 Hz (1 mm amplitude) 6 Hz (1 mm amplitude) and 10 Hz (1 mm amplitude) vibration groups, respectively. Thus, the nuclei maturation of oocytes cultured with dynamic culture did not differ from the static culture group.

### Effect of vibration stimulation on embryo compaction at 1.5 days

**Figure 3:**
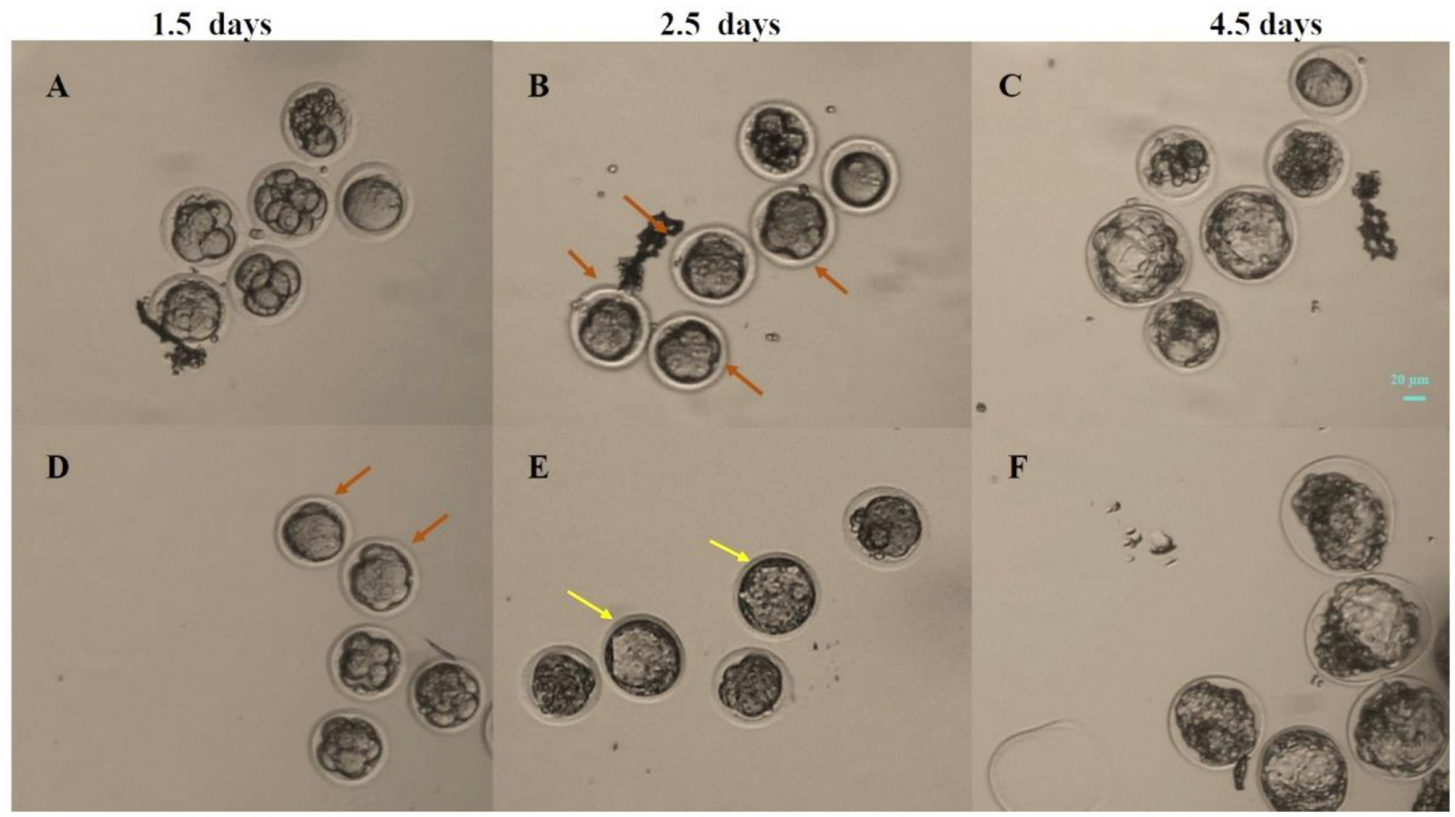
Photos of embryos at 1.5, 2.5 and 4.5 days under different culture conditions. **(A–C)** Static culture; **(D–F)** Vibration culture with an amplitude of 0.1 mm. The orange arrows indicate compacted embryos, while the yellow arrows indicate early blastocysts that appeared in the vibration stimulation group at 2.5 days. Scale bar: 20 μm.

The compaction of embryos was assessed at 1.5 days (Figures 3A, D) and 2.5 days (Figures 3B, E). The formation of blastocysts was observed at 3.5 and 4.5 days. Figure 3C showed a blastocyst formed under static cultured at 4.5 days, while Figure 3F showed a blastocyst formed after 3 Hz vibration culture. The number of compacted embryos and blastocysts was counted in each group, and the embryo compaction rate and blastocyst formation rate under different culture conditions were statistically analyzed.

**Figure 4:**
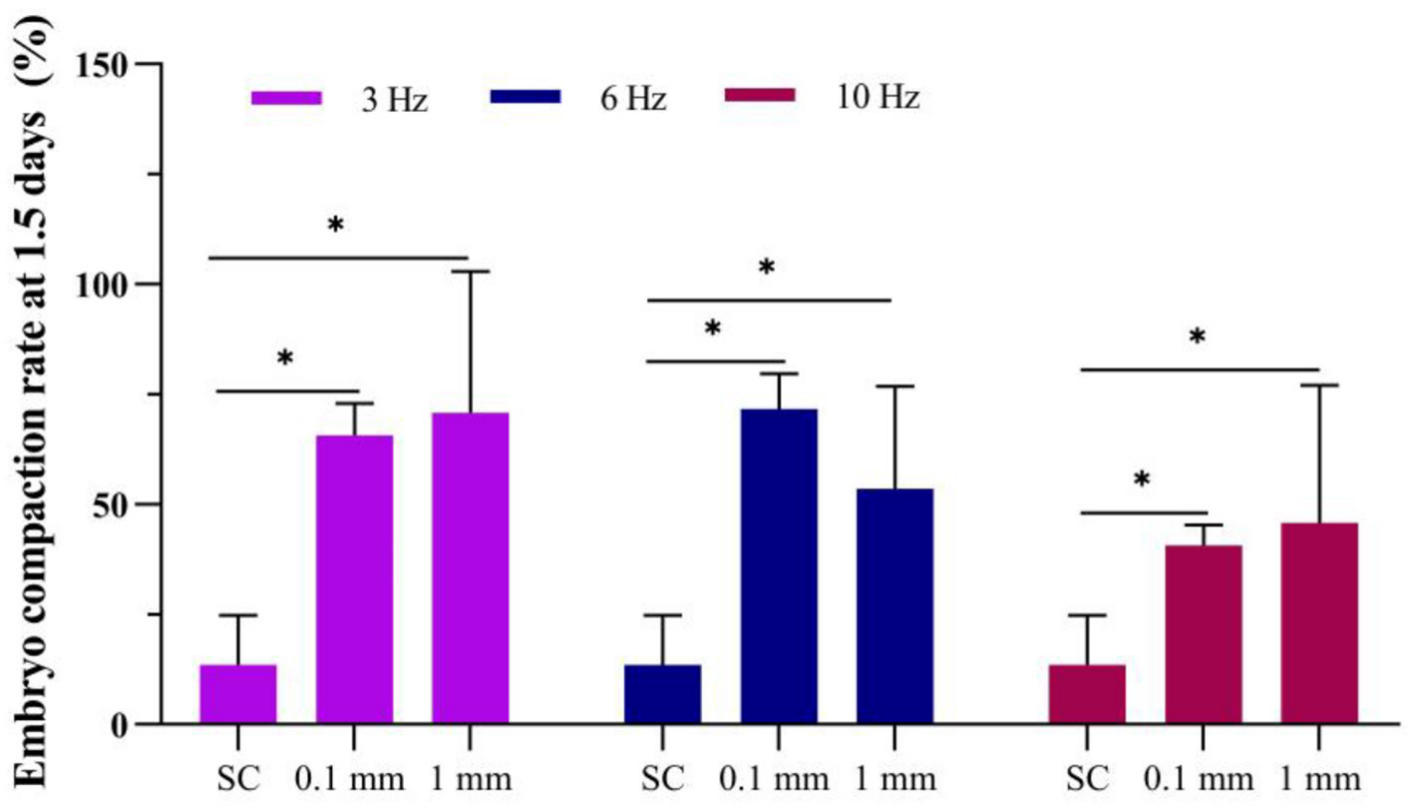
Effect of vibration stimulation on embryo compaction at 1.5 days. SC, Static culture; 1 mm: Amplitude is 1 mm; 0.1 mm: Amplitude is 0.1 mm; 3 Hz, 6 Hz, 10 Hz represent vibration frequencies. Error bars are SEM. *P < 0.05.

The results showed that at 1.5 days, the embryo compaction rate in the static culture group was 13.6%. Under an amplitude of 0.1 mm, the embryo compaction rates for 3 Hz, 6 Hz, and 10 Hz culturing methods were 65.7%, 71.7%, and 40.6%, respectively (Figure 4). Under an amplitude of 1 mm, the embryo compaction rates for 3 Hz, 6 Hz, and 10 Hz culturing methods were 70.8%, 53.6% and 45.8%, respectively. The embryo compaction rates at 1.5 days in the dynamic culture groups were significantly higher than those in the static culture group.

### Effect of vibration stimulation on embryo compaction at 2.5 days

**Figure 5:**
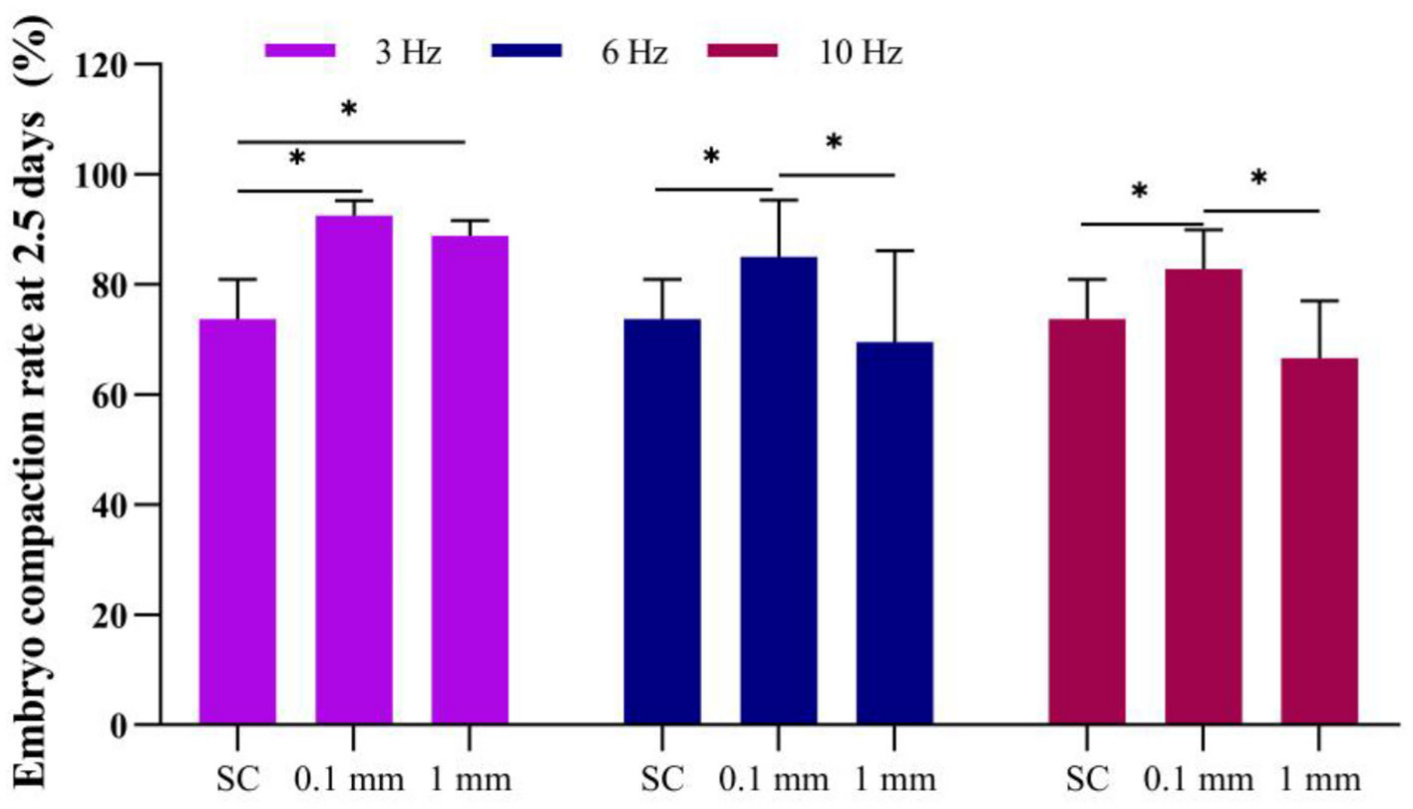
Effect of vibration stimulation on embryo compaction at 2.5 days. SC, Static culture; 1 mm: Amplitude is 1 mm; 0.1 mm: Amplitude is 0.1 mm; 3 Hz, 6 Hz, 10 Hz represent vibration frequencies. Error bars are SEM. *P < 0.05.

When the embryos developed to day 2.5, the results showed that in the static culture group, the embryo compaction rate was 73.8%. Under an amplitude of 0.1 mm, the embryo compaction rates for 3 Hz, 6 Hz, and 10 Hz vibrational culture group were 92.6%, 85.0%, and 82.8%, respectively, all of which were significantly higher than the static culture group (Figure 5).

Under an amplitude of 1 mm, the embryo compaction rates for 3 Hz, 6 Hz, and 10 Hz culturing methods were 88.9%, 69.6%, and 66.7%, respectively. The embryo compaction rates rate at a frequency of 3 Hz was significantly higher than that of the static culture group; when the frequency was 6 Hz and 10 Hz, there was no significant difference compared to static culture (Figure 5).

This result indicates that when the vibration amplitude is the same, the compaction rate of embryos at 2.5 days in the low-frequency group is significantly higher than that in the high-frequency group. When the vibration frequency was the same, the compaction rate of embryos in the low-amplitude group was significantly higher than that in the high-amplitude group. This suggested that applying continuous vibration stimulation to embryos at a lower frequency and lower amplitude can significantly increase the compaction rate of embryos and accelerate the compaction process.

### Effect of vibration stimulation on blastocyst formation

**Figure 6:**
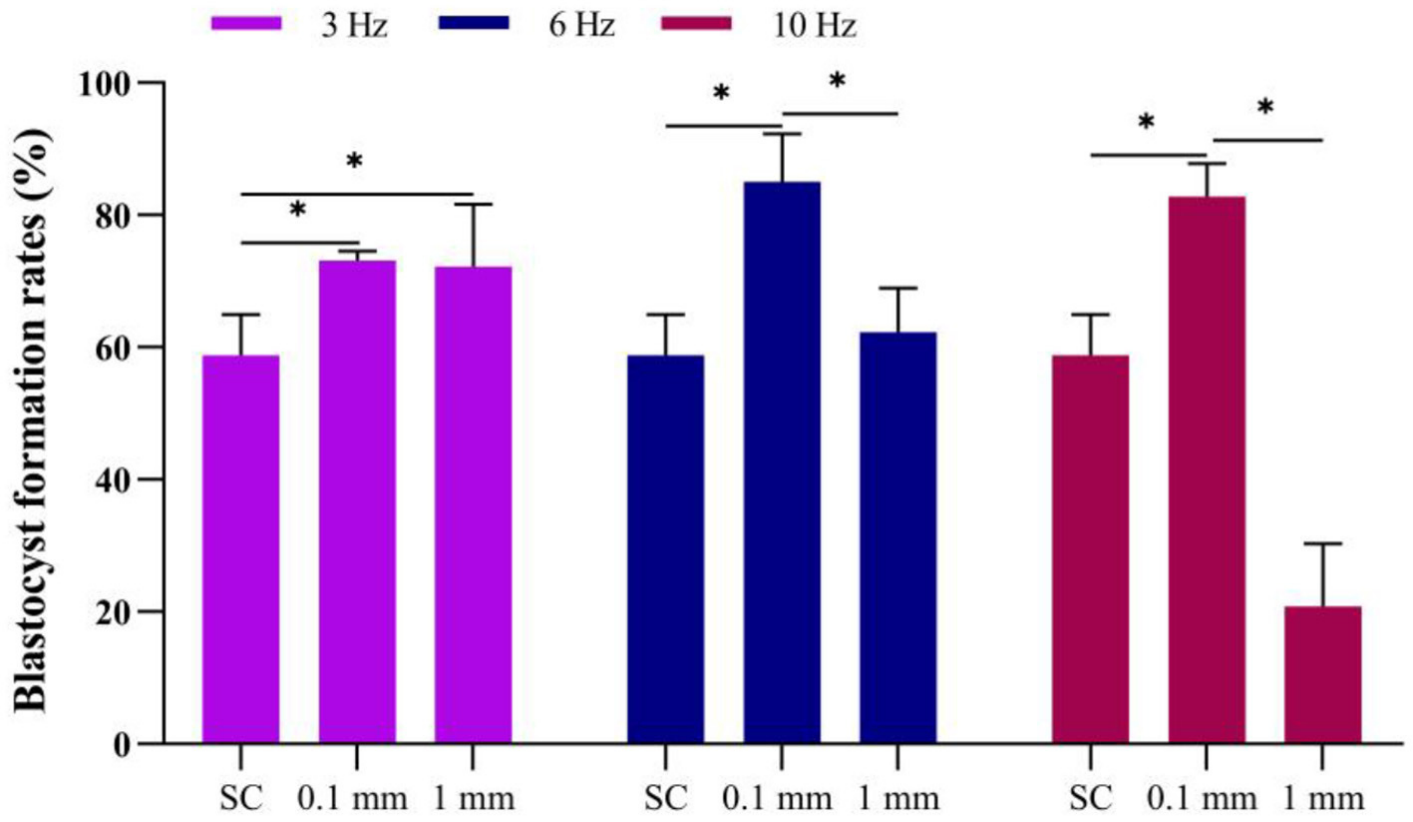
Effect of vibration stimulation on blastocyst formation. SC, Static culture; 1 mm: Amplitude is 1 mm; 0.1 mm: Amplitude is 0.1 mm; 3 Hz, 6 Hz, 10 Hz represent vibration frequencies. Error bars are SEM. *P < 0.05.

Under an amplitude of 0.1 mm, the blastocyst formation rates for the 3 Hz, 6 Hz, and 10 Hz dynamic culture methods were 73.2%, 85.0% and 82.8%, respectively, all of which were significantly higher than the static culture group. Under dynamic culture conditions at a vibration frequency of 3 Hz, there was no difference (*P* = 0.512) in blastocyst formation rates between the groups with 0.1 mm and 1 mm amplitudes. However, at frequencies of 6 Hz (*P* = 0.005) and 10 Hz (*P* = 0.000), the blastocyst formation rate in the 0.1 mm amplitude group was significantly higher than that in the 1 mm amplitude group (Figure 6).

Under an amplitude of 1 mm, the blastocyst formation rates for the 3 Hz, 6 Hz, and 10 Hz culture methods were 72.2%, 62.3% and 20.8%, respectively. The blastocyst formation rate at a frequency of 3 Hz was significantly higher than that of the static culture group; When the frequency was 6 Hz, there was no significant difference compared to static culture (*P* = 0.674). However, at a frequency of 10 Hz, the blastocyst formation rate was significantly reduced (*P* = 0.000) (Figure 6).

### Numerical simulation of FSS of oocytes vibrating dynamic culture system

**Figure 7:**
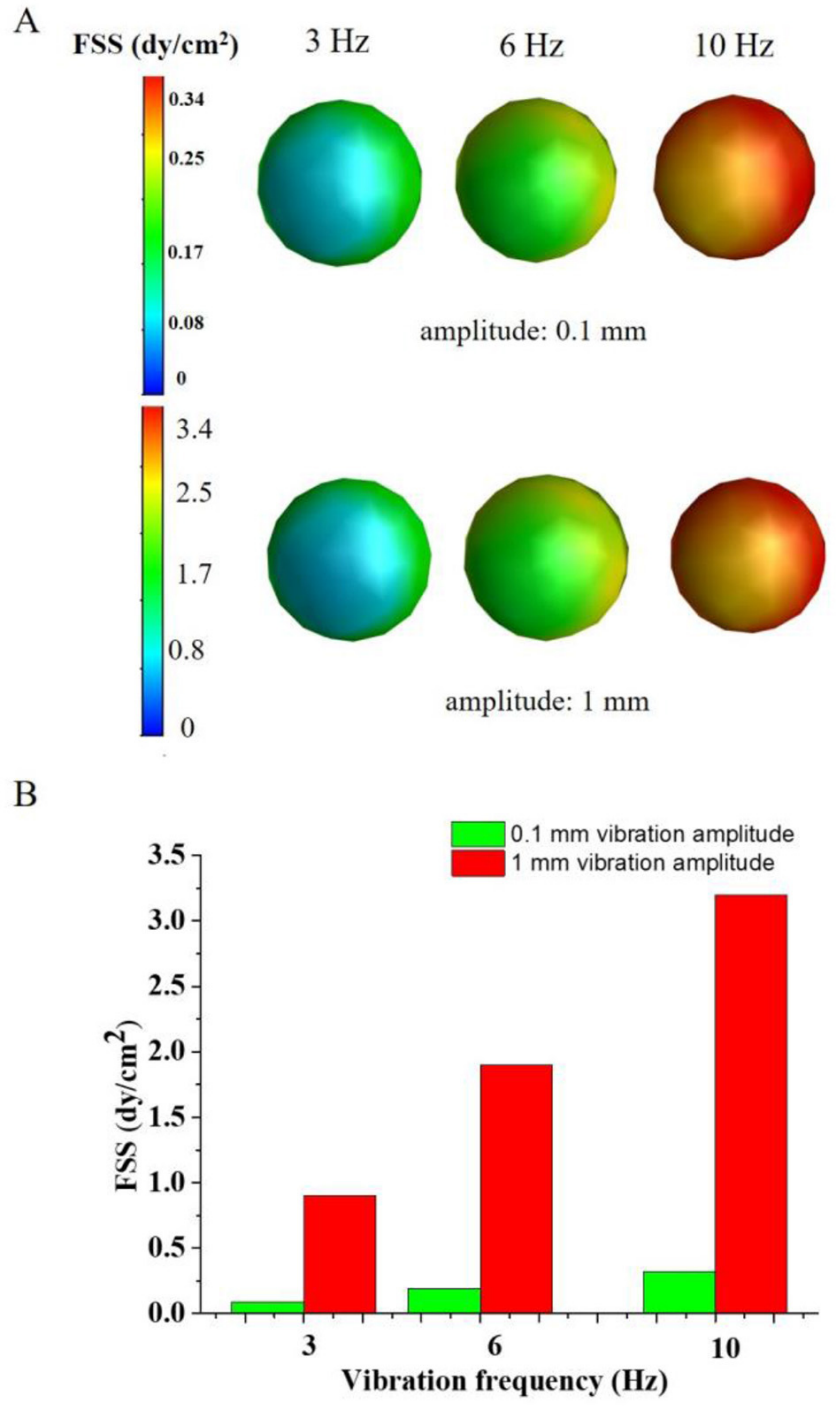
The FSS distribution of the oocytes. **(A)** the FSS profile of the oocytes; **(B)** the average FSS on the oocytes under different vibration frequency and amplitude.

Figure 7 showed that the FSS of oocytes vibrating dynamic culture system. The FSS of oocytes were 0.9 dyne/cm^2^, 1.9 dyne/cm^2^ and 3.2 dyne/cm^2^ corresponding to the 3 Hz, 6 Hz and 10 Hz vibration frequency with the 1 mm vibration amplitude, respectively. When the vibration amplitude reduced to 0.1 mm, the FSS of oocytes decreased to 0.09 dyne/cm^2^, 0.19 dyne/cm^2^ and 0.32 dyne/cm^2^, respectively. It can be seen that the higher vibration amplitude and vibration frequency produced higher FSS on the oocytes (Figure 7B). Compared with the experimental results, higher FSS will change the mechanical microenvironment of the oocytes, which may lead to changes in the physiological function of the oocytes.

### Numerical simulation of stiffness of oocytes under vibrating stimulation

When the FSS of oocytes was 0.9 dyne/cm^2^, the oocyte principle stress was ~1.3 Pa. Correspondingly, when the FSS of oocytes increased to 3.2 dyne/cm^2^, the oocytes principle increased to 4.5 Pa. The simulation results indicated a linear relationship between the FSS level and the stress experienced by oocytes.

**Figure 8:**
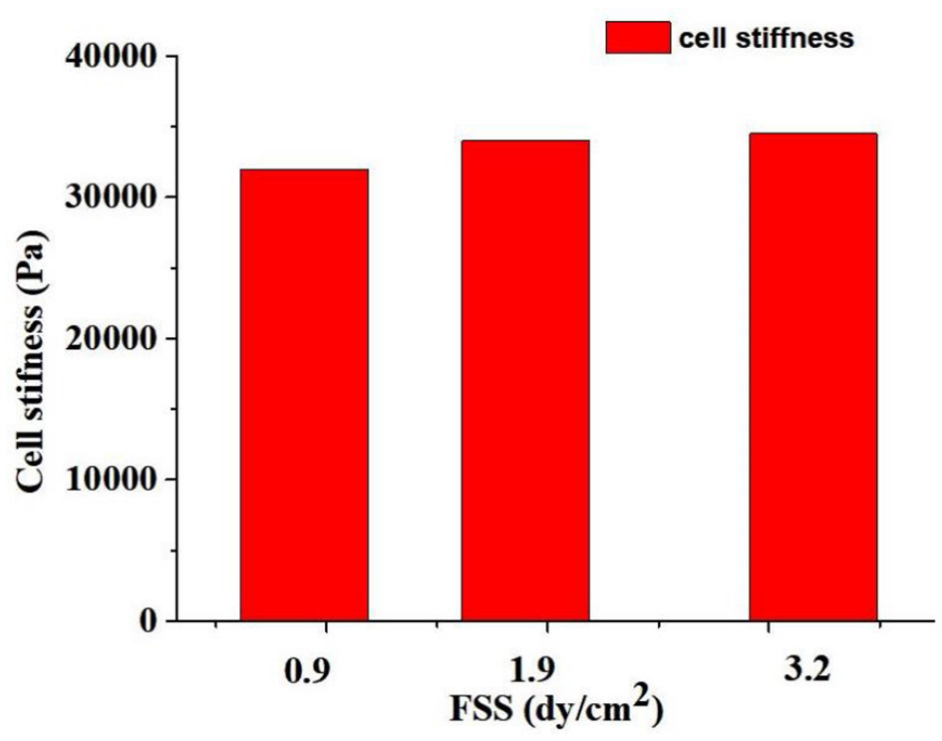
The oocytes stiffness effected by the different FSS.

Figure 8 showed that the calculated stiffness of oocytes under vibrating stimulation. The stiffness of oocytes was 32,000, 34,000, and 34,500 Pa when the FSS were 0.9 dyne/cm^2^, 1.9 dyne/cm^2^ and 3.2 dyne/cm^2^, respectively. It can be seen that the higher FSS caused larger stiffness of oocytes, which indicated that the FSS changes the oocytes stiffness and affects the oocytes growth and development.

## Discussion

In present study, the applied appropriate vibration frequencies and amplitudes were obtained for determination of device parametric variables to facilitate the oocytes maturation and subsequential developmental potential. Through the compared results of the blastocyst formation rate, the external mechanical vibration stimulation could vary the cellular stiffness, and sequentially promote the parthenogenetic development of mouse oocytes. Previously, Mizobe et al. (8) applied vibration at 20 Hz per min for 5 s to immature pig oocytes and found a significant increase in their maturation and parthenogenetic developmental potential (8). That study used a relatively high frequency and short vibration duration. However, we considered that the ciliary beating continuously at a low frequency during the *in vivo* process (13, 18). Therefore, the continuous vibration stimulation of 3 Hz and 6 Hz was used in this study. The mechanical loading cases of 3 Hz (0.1 mm amplitude), 6 Hz (0.1 mm amplitude), 10 Hz vibration (0.1 mm amplitude) and 3 Hz vibration (1 mm amplitude) were found to significantly increase the compaction ratio of embryos, and to improve the rate of blastocyst formation, i.e. improve the developmental potential of the immature oocytes in mouse. However, the 6 Hz to 10 Hz vibration (1 mm amplitude) significantly decreased the developmental potential of embryos. The results of the numerical simulation showed that higher FSS caused larger stiffness of oocytes, which indicated that the FSS changes the oocytes stiffness and affects the *in vitro* developmental potential of oocytes. We hypothesize that under the two loading methods (6 Hz and 10 Hz vibration with 1 mm amplitude), the FSS (≥ 2.0 dyne/cm^2^)around the cells and the cell stiffness (≥34,500 Pa) exceeded the normal values, ultimately leading to a decline in the developmental potential of the oocytes.

The cell adhesion molecule E-cadherin is thought to mediate the intercellular adhesion that starts the process of cell compaction (19, 20). In the present study, we found that dynamic culture promoted more embryos developing to the compacted embryo stage (Figures 4, 5). We hypothesize that mechanical stimulation of early embryos can induce changes in the location and morphology of mechanoreceptors on the cell membrane (21). Consequently, transmitting mechanical signals from the extracellular space to the intracellular compartment causes a force response in the embryo that induces the expression of E-cadherin, promoting cell compaction.

The main problem with *in vitro* mature oocytes is the lack of synchronization between the nucleus and cytoplasmic maturation. Mizobe et al. showed that microvibration stimulation in culture could improve the cytoplasmic maturation of porcine oocytes (8). Oocytes maturation includes nuclear and cytoplasmic maturation, and the cytoskeleton plays an important role in the maturation of oocytes. The cytoskeleton is involved in oocyte spindle migration, oocyte polarization, polar body expulsion, cytoplasmic division, and organelle rearrangement (22, 23). In addition to maintaining and changing the morphology of cells and the positioning of organelles, the cytoskeleton can also conduct and sense changes in the mechanical environment in which it is placed. The cytoskeleton senses the changes of mechanical environment and cells transmits mechanical signals between cells, regulating the life activities of cells (24). Thus, we hypothesize that this increase in developmental potential may be due to the transmission of mechanistic signals from the cytoskeleton to the cell, causing changes in the distribution and organization of F-actin and tubulin, inducing an intracellular response that regulates the maturation of oocytes, including cytoplasmic maturation and nuclear maturation, and thus increases their developmental potential.

For the limitation of this study, several groups of device vibration parameters were applied for the comparison study, thus, more mechanical loading variables would be explored by parametric optimization to promote the parthenogenetic development of oocytes. Additionally, the proposed numerical modeling only simulated one vibration cycle for exploring the mechanical influences on cell maturation. The impacting mechanisms of fluid filed were analyzed based on the unidirectional fluid-solid coupling simulation. In our future work, bidirectional fluid-solid coupling simulation would be adopted to systematically investigate the influences of cell stiffness on the mechanical properties of fluid fields.

## Conclusion

This study concluded that optimizing the embryo culture system requires considering the mechanical environment in which the cells develop. In addition to chemical factors, mimicking the *in vivo* growth and developmental environment is crucial. This study found that the mechanical stimulation of immature mouse oocytes enhances their parthenogenetic developmental potential, providing a novel approach to improve the IVM (*in vitro* maturation) efficiency and obtaining parthenogenetic embryonic stem cells from immature oocytes.

## Data Availability

The original contributions presented in the study are included in the article/supplementary material, further inquiries can be directed to the corresponding authors.
